# Influence of Delivery Method on Neuroprotection by Bone Marrow Mononuclear Cell Therapy following Ventral Root Reimplantation with Fibrin Sealant

**DOI:** 10.1371/journal.pone.0105712

**Published:** 2014-08-26

**Authors:** Roberta Barbizan, Mateus V. Castro, Benedito Barraviera, Rui S. Ferreira, Alexandre L. R. Oliveira

**Affiliations:** 1 Laboratory of Nerve Regeneration, Department of Structural and Functional Biology, University of Campinas - UNICAMP, Campinas, São Paulo, Brazil; 2 Center for the Study of Venoms and Venomous Animals (CEVAP), São Paulo State University (UNESP – Univ Estadual Paulista), Botucatu, São Paulo, Brazil; Wake Forest Institute for Regenerative Medicine, United States of America

## Abstract

The present work compared the local injection of mononuclear cells to the spinal cord lateral funiculus with the alternative approach of local delivery with fibrin sealant after ventral root avulsion (VRA) and reimplantation. For that, female adult Lewis rats were divided into the following groups: avulsion only, reimplantation with fibrin sealant; root repair with fibrin sealant associated with mononuclear cells; and repair with fibrin sealant and injected mononuclear cells. Cell therapy resulted in greater survival of spinal motoneurons up to four weeks post-surgery, especially when mononuclear cells were added to the fibrin glue. Injection of mononuclear cells to the lateral funiculus yield similar results to the reimplantation alone. Additionally, mononuclear cells added to the fibrin glue increased neurotrophic factor gene transcript levels in the spinal cord ventral horn. Regarding the motor recovery, evaluated by the functional peroneal index, as well as the paw print pressure, cell treated rats performed equally well as compared to reimplanted only animals, and significantly better than the avulsion only subjects. The results herein demonstrate that mononuclear cells therapy is neuroprotective by increasing levels of brain derived neurotrophic factor (BDNF) and glial derived neurotrophic factor (GDNF). Moreover, the use of fibrin sealant mononuclear cells delivery approach gave the best and more long lasting results.

## Introduction

In order to enhance the success of adult stem cell (SC) translational medicine efforts, the source as well as the most effective delivery method has to be considered. The bone marrow contains endothelial progenitor cells and mononuclear cells (MC). The MC fraction corresponds to the totality of hematopoietic and mesenchymal stem cells. MC present clinical advantages over other stem cells, based on the minimally invasive harvesting procedures, which are fast and cost-effective. Also, the possibility of autografting avoids the use of immunosuppressants, present low oncogenic potential and does not raise ethical issues [1] as compared to other SC. Moreover, MCs have similar potential therapeutic outcome for nerve regeneration in comparison to mesenchymal cells [2]. The peripheral nerve regeneration after MC has been connected to the local production of neurotrophic factors [1], [3], [4]. Relevantly, stem cell therapy may also present an immunomodulatory effect, reducing pro-inflammatory events as well as glial reaction following lesion.

Ventral root avulsion in rats has been used as a model for brachial plexus lesion (BPL). BPL is frequently caused by motorbike accidents in young adults as well as following complicated child-birth delivery [5]. It causes paralysis in the corresponding muscle groups and loss of sensory functions [6]. The degenerative impact on motoneurons is well characterized and is potentiated by pulling out the ventral roots from the CNS/PNS interface at the spinal cord surface [6]. Similarly to BPL, VRA results in extensive loss of neurons in the first weeks after injury [7], [8].

Reimplantation of avulsed roots can rescue motoneurons from degeneration, increasing the regenerative capacity of axonal regrowth [9], [10]. As a result, anatomical and functional reinnervation of denervated muscles can be obtained [11]–[13]. As seen in a previous work [10], a snake venom derived fibrin sealant allowed successful and stable ventral root implantation. Nevertheless, additional therapeutic approaches need to be developed, since root reimplantation alone, although neuroprotective, results in insufficient functional sensory-motor recovery [12], [14]–[16].

In order to improve the outcome following VRA, regarding neuronal survival, several attempts have been made to provide neurotrophic molecules at the site of injury. In this regard, the association of the root reimplantation with BDNF and CNTF resulted in rescue of injured motoneurons after avulsion in rabbits [17]. Therefore, the use of neurotrophic factors in combination with root reimplantation is a potential therapy to be used in patients.

The use of recombinant neurotrophic factors, however, present important drawbacks. One of them is the need of relatively large amounts of the purified substance, to reach the target lesioned area. Due to the short biological activity window of such substances, there is also need of constant perfusion, what may contribute to infection and further lesion of the affected spinal cord area. Additionally, it is improbable that a single neurothrophic molecule will be sufficient to provide the necessary conditions for optimal regeneration.

Based on such facts, the advent of stem cell technology brought new insights on cell therapy and local delivery of trophic substances. To date, however, there is not sufficient data on the delivery method to the nervous system, especially following VRA. So far, it is known that mesenchymal stem cells synthesize and possibly release BDNF and GDNF, when grafted to the VRA lesion area [18]. No data, however, indicates that MC exhibit the same properties.

Therefore, the present study investigated two delivery strategies of MC, comparing the local injection to the spinal cord with the possibility of mixing MC with fibrin sealant on the interface of the CNS/PNS. Local production of BDNF and GDNF were evaluated in both situations.

The results herein demonstrate that MC therapy is neroprotective and increases the transcript and protein levels of BDNF and GDNF in the lesioned spinal cord area. Moreover, the administration method significantly influenced treatment outcome, so that the use of fibrin sealant MC delivery approach gave the best and more long lasting results.

## Material and Methods

### 2.1- Experimental animals

Adult female Lewis (LEW/HsdUnib) rats, 7 weeks old, were obtained from the Multidisciplinary Center for Biological Investigation (CEMIB/UNICAMP) and housed under a 12-hour light/dark cycle with free access to food and water. The study was approved by the Institutional Committee for Ethics in Animal Experimentation (Committee for Ethics in Animal Use – Institute of Biology - CEUA/IB/UNICAMP, proc. n° 2073-1). All experiments were performed in accordance with the guidelines of the Brazilian College for Animal Experimentation. The animals were subjected to unilateral avulsion of the L4-L6 lumbar ventral roots and divided into 4 groups: 1) VRA without reimplantation (AV 1 week, n = 5; AV 4 weeks, n = 15 and AV 8 weeks, n = 10); 2) VRA followed by lesioned roots reimplantation with fibrin sealant (AV+S 1 week n = 5, AV+S 4 weeks, n = 15 and AV+S 8 weeks, n = 10); 3) VRA followed by lesioned roots reimplantation with fibrin sealant and MC homogenized to the sealant (AV+S+HC 1 week, n = 5, AV+S+HC 4 weeks, n = 15 and AV+S+HC 8 weeks, n = 10) and 4) VRA followed by lesioned roots reimplantation with fibrin sealant and injection of MC (AV+S+IC 4 weeks, n = 15 and AV+S+IC 8 weeks, n = 10).

The peroneal functional index was calculated weekly up to 8 weeks after injury (n = 10 for each group). Animals were killed 1 week and 4 weeks after injury and their lumbar spinal cords were processed for PCR (n = 5 for each group). The animals were killed after 4 and 8 weeks after avulsion and the lumbar spinal cords were processed for immunohistochemistry (n = 5 for each group) and neuronal survival counting (n = 5 for each group). Each animal's unlesioned, contralateral spinal cord side, served as an internal control.

### 2.2- Bone Marrow Mononuclear Cells Extraction

MC were extracted from transgenic Lewis rats (LEW-Tg EGFP F455/Rrrc), with the EGFP (Enhanced green fluorescent protein) gene under Ubiquitin C promoter control. The animals were imported from Missouri University (EUA) and were provided by Dr. Alfredo Miranda Góes, Federal University of Minas Gerais – UFMG, Brazil.

The EGFP rats were killed with lethal dose of halothane (Tanohalo, Cristália Chemicals and Pharmaceuticals, Brazil), and the femur and tibia were dissected out from the muscular and connective tissue. The cells were isolated by density gradient centrifugation using Histopaque 1077 following the separation of mononuclear cells methods of Sigma-Aldrich n° 1119 protocol. The cell suspension consists of heterogeneous cell populations (13.2% CD11b, 1.52% CD3, 92.2% CD45, 20.8% CD34 of CD45). After 2 washing steps, cells were immediately transplanted.

### 2.3- Ventral root avulsion (VRA)

The rats were anesthetized with 50 mg/Kg of Ketamine (Fort Dodge) and 10 mg/Kg of xylasine (Köning) and subjected to unilateral avulsion of the lumbar ventral roots as previously described [19]. Right side avulsion was performed at the L4, L5 and L6 lumbar ventral root after laminectomy. A longitudinal incision was made to open the dural sac, the denticulate ligament was dissected and the ventral roots associated with the lumbar intumescence could be identified and avulsed with fine forceps (№ 4). Finally, the musculature, fascia and skin were sutured in layers. Chlorhydrate of tramadol was administrated by gavage after the surgical procedures (20 mg/kg) and 2.5 mg/day soluble in water during 5 days.

### 2.4- Reimplantation of the motor roots

In AV+S, AV+S+MC and AV+S+IC groups, the roots were replaced at the exact point of detachment, on the ventral surface of the lumbar spinal cord at the avulsion site with the aid of a snake venom fibrin sealant [10]. The fibrin sealant was kindly provided by CEVAP/UNESP and is under the scope of the Brazilian Patents BR 10 2014 011432 7 and BR 10 2014 011436 0 [20]–[22]. The sealant used herein is composed of three separate solutions (1-fibrinogen, 2-calcium chloride and 3-thrombin-like fraction). During surgical repair of the avulsed roots, the first two components were applied and the avulsed roots were returned to their original sites. The third component was then added for polymerization. The reimplanted roots were then gently pulled from the spinal cord, and the stability of the fixation was observed to evaluate the success of the repair.

### 2.5- Mononuclear cells transplantation

**Figure 1:**
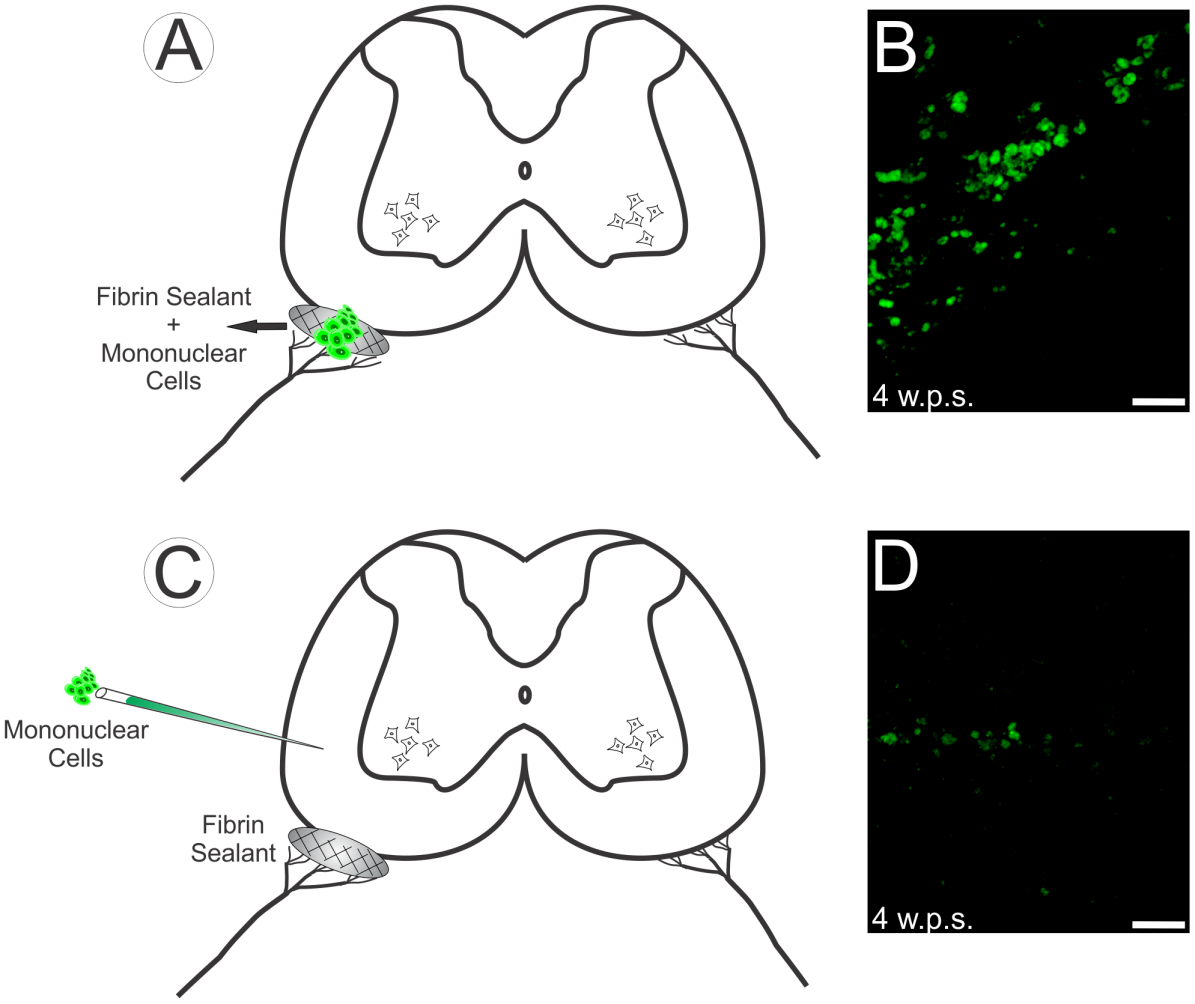
Scheme of the spinal cord subjected to motor root avulsion and reimplantation, with the two different cell delivery locations. A) Scheme of transverse spinal cord section subjected to motor avulsion followed by fibrin sealant reimplantation plus MC. B) MC are grouped in the right ventrolateral surface of the spinal cord (AV+S+HC), four weeks post surgery (4 w.p.s.). C) Scheme of the transverse spinal cord section subjected to motor root avulsion followed by fibrin sealant reimplantation and cell injection in the lateral funiculus. D) The cells are at the site of injection (AV+S+IC), 4 w.p.s. Such cells were E-GFP fluorescent donors. Scale bar = 50 µm.

In group 3 (AV+S+HC), 3×10^5^ MCs were added to the fibrin sealant in avulsed roots reimplantation at the moment of implantation. In this case, the cells are grouped in the right ventrolateral surface of the spinal cord (Figure 1 A and B). In group 4, 3×10^5^ MCs were placed directly into the lateral funiculus on the lesioned side (ipsilateral) in the segments L4–L6 of the spinal cord with the aid of a thick capillary Pasteur pipette coupled to a Hamilton syringe and then the avulsed roots were repaired with sealant. In this group, the cells are at the site of injection (Figure 1 C and D).

### 2.6- Specimen preparation

The animals were anaesthetized with an overdose of mixture of xylasine and Ketamine, and the vascular system was transcardially perfused with phosphate buffer 0.1 M (pH 7.4). For PCR, the rats were killed 1 and 4 weeks after VRA and their lumbar intumescences were frozen in liquid nitrogen. For neuron survival counting and immunohistochemistry the rats were killed 4 and 8 weeks after VRA and fixed by vascular perfusion of 10% formaldehyde in phosphate buffer (pH 7.4). The lumbar intumescence was dissected, post-fixed overnight and then washed in phosphate buffer and stored overnight in sucrose 20% before freezing. Transverse cryostat 12 µm thick sections of spinal cords were obtained and transferred to gelatin-coated slides and dried at room temperature for 30 min before being stored at −20°C.

### 2.7- Counting of motoneurons survival

Cell counts were performed on sections from the lumbar enlargement. Transverse cryostat sections of the spinal cords were stained for 3 min in aqueous 1% cresyl fast violet solution (Sigma-Aldrich, USA). The sections were then washed in distilled water, dehydrated and mounted with Entellan (Merck, USA). The motoneurons were identified (based on their morphology, size, and location in the dorsolateral lamina IX) and cells with a visible nucleus and nucleolus were counted. The absolute number of motoneurons on the lesioned and non-lesioned side of each section was used to calculate the percentage of surviving cells in each specimen. This percentage was calculated by dividing the number of motoneurons in the ipsilateral (lesioned) side by the number of neurons in the contralateral (non-lesioned) side and multiplying the result by 100. Abercrombie's formula was used to correct for the duplicate counting of neurons [23]. 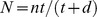
where *N* is the corrected number of counted neurons, *n* is the counted number of cells, *t* is the thickness of the sections (12 µm) and *d* is the average diameter of the cells. Because differences in cell size can significantly affect cell counts, the value of *d* was calculated specifically for each experimental group and for both ipsilateral and contralateral neurons. The diameters of 15 randomly chosen neurons from each group were measured by using the ImageTool software (version 3.00, The University of Texas Health Science Center, San Antonio, USA).

### 2.8- Immunohistochemistry

Transverse sections of spinal cord were incubated with mouse anti-synaptophysin (Dako, 1∶250), goat anti-GFAP (Dako, 1∶900), and rabbit anti-Iba1 (Wako, 1∶800) diluted in a solution containing 1% BSA in TBS-T (Tris-Buffered Saline and Tween) and 2% Triton X-100 in PB 0.1 M (phosphate buffer). All sections were incubated for 6 hours at room temperature in a moist chamber. After rinsing in TBS-T, the sections were incubated according to the primary host antibody (CY-3, Jackson Immunoresearch; 1∶250) for 45 minutes in a moist chamber at room temperature. After 3 times washing in TBS-T, the slides were mounted in a mixture of glycerol/PBS (3∶1) and observed with a Nikon eclipse TS100 inverted microscope (Nikon, Japan). For quantitative measurements, 3 representative images (with 2 MNs) of the spinal cord (L4–L6 at lamina IX, ventral horn) from each animal were captured at a final magnification of ×200. Double blind quantification was performed in IMAGEJ software (version 1.33 u, National Institute of Health, USA) using the enhanced contrast and density slicing two features [24]. The integrated density of pixels was systematically measured in six representative areas of the motor nucleus from each section, according to [19]. The integrated pixel density was calculated for each section of spinal cord, and then a mean value for each spinal cord was calculated. The data are represented as the mean ± standard error (SE).

### 2.9- Real time polymerase chain reaction (PCR)

Total RNA was extracted from the ipsilateral and contralateral sides of the frozen lumbar intumescences, 1 and 4 weeks after lesion, using the RNeasy Lipid Tissue Kit (cat n° 74804, Quiagen), according to the manufacturer's recommendations. The RNA was quantified using a NanoDrop Spectrophotometer (A260/280; model 2000, Thermo Scientific). The RNA (1 µg) obtained from five samples was reverse-transcribed using a commercial kit (AffinityScripts QPCR cDNA Synthesis Kit - Agilent Technologies, La Jolla, CA, USA) to achieve a final reaction volume of 20 µL. Real time quantitative PCR was performed on Mx3005P qPCR System (Agilent Technologies, La Jolla, CA, USA), after an initial denaturation for 10 minutes at 95°C, followed by 35 cycles of amplification (95°C for 30 seconds followed by 72°C for one minute). The reactions were carried out with 12.5 µL 2×SYBR Green PCR master mix (Agilent Technologies), 0.2 µM of each forward and reverse primer 100 ng cDNA template, in a final reaction volume of 20 µL. All quantifications were normalized to the house keeping gene β-actin. A non-template control with non-genetic material was included to eliminate contamination or nonspecific reactions. Each sample (n = 5) was tested in triplicate and then used for the analysis of the relative transcription data using the 2^−ΔΔCT^ method [25]. The following forward (F) and reverse (R) primers were used: BDNF: (F) 5′-CCACAATGTTCCACCAGGTG-3′, (R) 5′-TGGGCGCAGCCTTCAT-3′; GDNF: (F) 5′-CCACCATCAAAAGACTGAAAAG-3′, (R) 5′-CGGTTCCTCTCTCTTCGAGGA-3′; Iba-1 (F) 5′- CCCCACCTAAGGCCACCAGC-3′, (R) 5′-TCCTGTTGGCTTTCAGCAGTCC-3′; GFAP (F) 5′-TGCTGGAGGGCGAAGAAAACCG-3′ 5′-CCAGGCTGGTTTCTCGGATCTGG-3′; β-actin (F) 5′-GGAGATTACTGCCCTGGCTCCTA-3′ (R) 5′-GACTCAICGTACTCCTGCTTGCTG-3′. The BDNF and GDNF were based on [18] and the β-actin was based on [26].

### 2.10- Functional Analysis

For the gait recovery analysis, the CatWalk system (Noldus Inc., The Netherlands; was used. In this set up, the animal crosses a walkway with an illuminated glass floor. A high-speed video camera Gevicam (GP-3360, USA) equipped with a wide-angle lens (8.5 mm, Fujicon Corp., China) is positioned underneath the walkway and the paw prints are automatically recorded and classified by the software. The paw prints from each animal were obtained before and after the VRA. Post-operative CatWalk data were collected twice a week for 12 weeks. The peroneal functional index (PFI) was calculated as the distance between the third toe and hind limb pads (print length) and the distance between the first and fifth toes (print width). Measurements of these parameters were obtained from the right (lesioned) and left (unlesioned) paw prints, and the values were calculated using the following formula [27]. 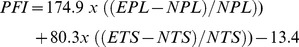



Where N: normal, or non-operated side; E: experimental, or operated; PL: print length; TS: total toe spread, or distance between first to fifth toe.

The pressure exerted on the platform by individual paws was also evaluated. The Catwalk data from each day were expressed as an ipsi-/contralateral ratio.

### 2.11- Statistical analysis

Statistical analysis was performed with Graphpad Prisma 4.0 software. The neuronal survival, immunohistochemistry, and PCR were firstly evaluated with one-way ANOVA. Data from the functional analysis was evaluated with two-way ANOVA. Bonferroni post-test was used to identify intergroup differences. The data are presented as the mean ± SE and the differences between groups were considered significant when the P-value was <0.05 (*), <0.01 (**) and <0.001 (***).

## Results

### 3.1- Neuroprotection after root reimplantation plus MC treatment after VRA

**Figure 2:**
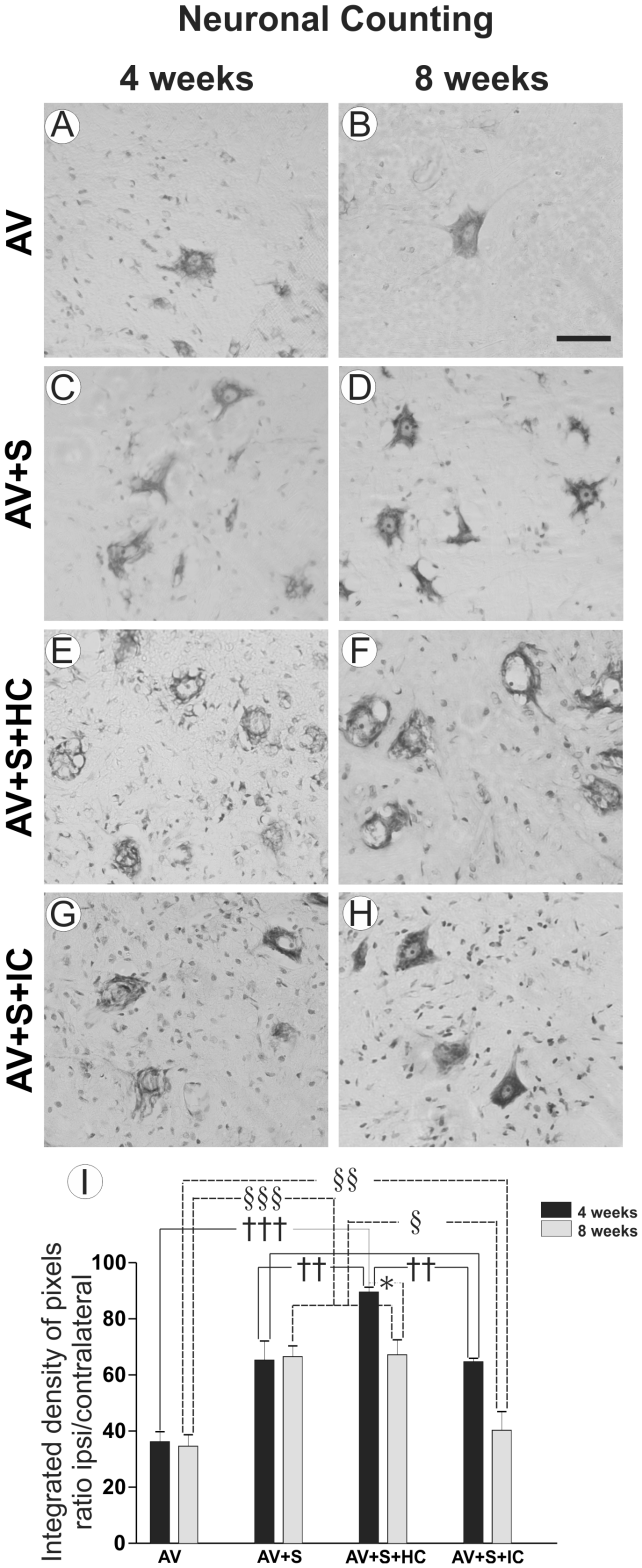
Nissl-stained spinal cord transverse sections at lamina IX illustrating the neuroprotective effects of root reimplantation and MC treatment on motoneurons 4 and 8 weeks after VRA. Motoneuron cell bodies of the ipsilateral side of VRA after 4 weeks (A, C, E and G) and after 8 weeks (B, D, F and H). (A and B) AV, (C and D) AV+S, (E and F) AV+S+HC and (G and H) AV+S+IC. Scale bar  = 50 µm. (I) Percentage of neuronal survival after ventral root avulsion, reimplantation and reimplantantion with MC. Note a significant rescue of lesioned neurons in the implanted groups with and without cells in two different survival times (4 and 8 weeks). This neuroprotection was even more intense in AV+S+HC 4 weeks after avulsion. (†† p<0.01 and ††† p<0.001 comparing all groups 4 weeks after avulsion, § p<0.05, §§ p<0.01 and §§§ p<0.001 comparing all groups 8 weeks after AV and *p<0.05 comparing AV+S+HC at two different times after avulsion, n = 5).

Neuronal survival was assessed as the ipsi-/contralateral ratio of motoneurons present in the lamina IX of the ventral horn. No significant differences between the numbers of motoneurons on the contralateral side in the different experimental conditions were observed. After four weeks, there was severe degeneration of affected motorneurons in the AV group (Fig. 2A). In implanted groups (Fig 2 C, E and G) a higher number of surviving neurons was observed. Such neuroprotection was even more evident in the AV+S+HC group (Fig. 2I) (AV 36.09%±3.67%; AV+S 65.19%±6.93%; AV+S+HC 89.44%±1.89%; AV+S+IC 64.60%±1.33% percentage of survival ipsi-/contralateral ± SEM; p<0.001).

Neuroprotection in the implanted groups remained superior to the avulsion only throughout the time course of the study, i.e. up to 8 weeks post lesion (Fig. 2 D, E and F). Nevertheless, the AV+S+HC group was similar to AV+S. Figure 2I shows the ipsi-/contralateral ratio for the groups where a statistically significant neuroprotective effect was observed (AV 34.58%±4.12%; AV+S 66.46%±3.88%; AV+S+HC 67.11%±4.83%; AV+S+IC 46.81%±5.87% percentage of survival ipsi-/contralateral ± SEM; p<0.001).

### 3.2- Decreased synaptic elimination after VRA followed by implantation and cell treatment

**Figure 3:**
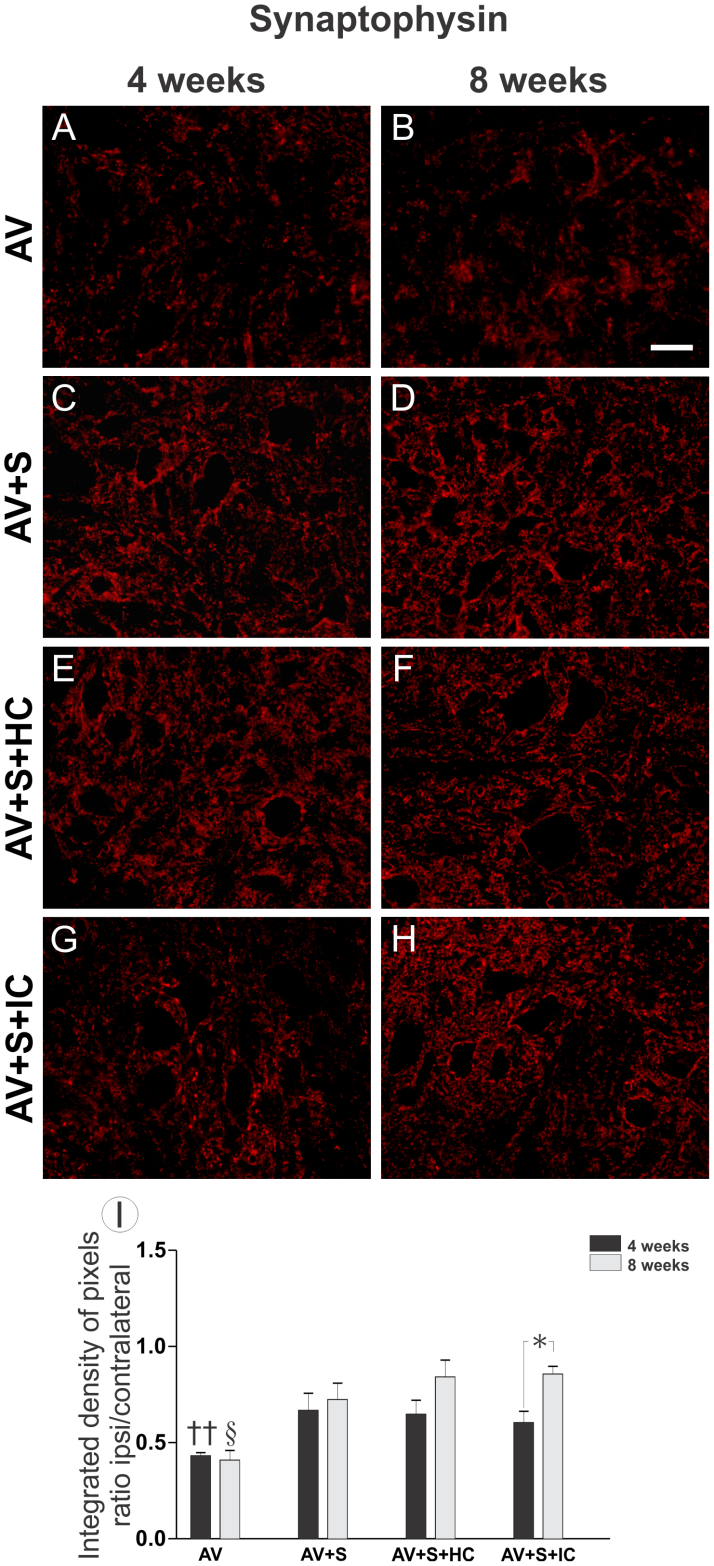
Immunohistochemical analysis of the spinal cord ventral horn stained with anti-synaptophysin 4 and 8 weeks after VRA. Observe the preservation of synaptophysin labeling, especially at the surface of the lesioned motoneurons in implanted group and both implanted with mononuclear cells treatment. After 4 weeks post lesion (A, C, E and G) and after 8 weeks (B, D, F and H). (A and B) AV, (C and D) AV+S, (E and F) AV+S+HC and (G and H) AV+S+IC. Scale bar  = 50 µm. (I) Quantification of synaptic covering obtained by the ratio ipsi/contralateral sides of the integrated density of pixels at lamina IX four and eight weeks after injury. (†† p<0.01 comparing all groups 4 weeks after avulsion, § p<0.05 comparing all groups 8 weeks after AV and * p<0.05 comparing AV+S+IC at two different times after avulsion, n = 5).

Synaptic network changes after root avulsion were evaluated in the ventral horn by immunohistochemistry with an antibody against synaptophysin. Quantitative measurements of synaptophysin immunoreactivity in the sciatic motor nuclei after avulsion (AV) and after avulsion followed by ventral root implantation (AV+S), implantation with cells homogenized on the sealant (AV+S+HC) and implantation with cell injection (AV+S+IC) were carried out. As shown in Fig. 3, AV only led to a significant decrease in synaptophysin expression four weeks after avulsion, which remained until 8 weeks. Such results indicate a significant decrease of complexity of intraspinal networks following lesion. In contrast, in implanted groups with or without cells, the repair resulted in preservation of synaptophysin immunoreactivity, in the immediate vicinity of the motoneurons. In AV+S+IC group the synaptic inputs decreased when comparing 4 and 8 weeks. Four weeks after avulsion: (AV 0.43±0.01; AV+S 0.66±0.09; AV+S+HC 0.65±0.07; AV+S+IC 0.60±0.01 mean ratio ipsi-/contralateral ± SE with p<0.01) and 8 weeks after avulsion: (AV 0.40±0.05; AV+S 0.72±0.05; AV+S+HC 0.84±0.07; AV+S+IC 0.855±0.03 mean ratio ipsi-/contralateral ± SE, p<0.05).

### 3.3- Astroglial reactivity is not further enhanced by mononuclear cells around motoneuron vicinity

**Figure 4:**
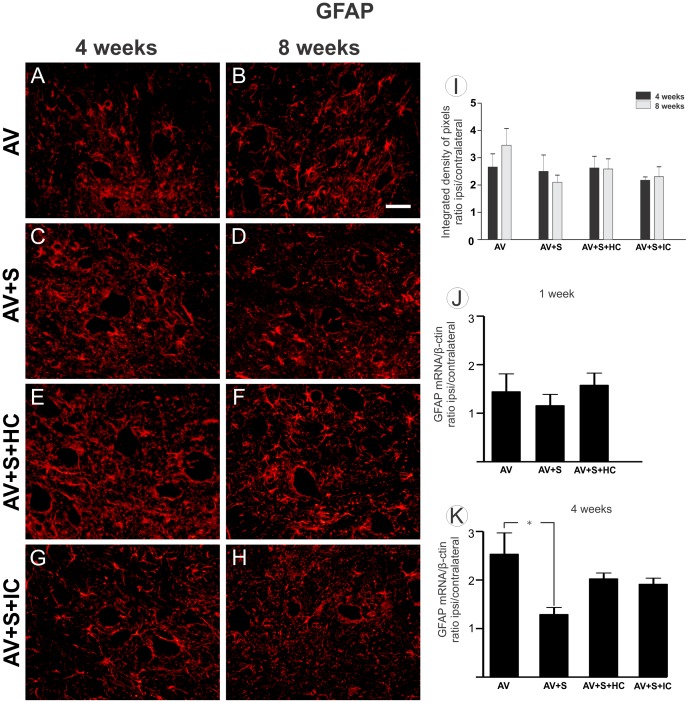
Glial fibrillary acidic protein (GFAP) in the spinal cord ventral horn. Immunohistochemical analysis of the anterior horn of the spinal cord was labeled with anti-GFAP, 4 and 8 weeks after injury to assess the degree of astroglial reactivity after root avulsion (A–H). Representative images of AV, AV+S, AV+S+HC and AV+S+IC. Scale bar  = 50 µm. Observe that ventral root implantation and cell treatment did not increase astroglial reaction. (I) The mean ratio of the ipsi-/contralateral integrated intensity of pixels of the ipsilateral and contralateral sides in all groups. (* p<0.05, n = 5). (J) One week after avulsion, there were no differences between groups by GFAPmRNA analysis. (K) The RT-qPCR performed 4 weeks after avulsion demonstrated significant decrease in the synthesis of GFAP mRNA in AV+S group compared with AV (*p<0.05, n = 5).

Immunoreactivity against GFAP was used to analyze the degree of astroglial reactivity after lesion. This demonstrates the presence of GFAP-positive astrocytic processes in the vicinity of the avulsed motoneurons. Figure 4 shows that the astroglial reactivity was not significantly further increased after implantation and cell treatment in both experimental times: Four weeks after avulsion (AV 2.65±0.48; AV+S 2.49±0.60; AV+S+HC 2.62±0.43; AV+S+IC 2.17±0.12 mean ratio ipsi-/contralateral ± SE); eight weeks after avulsion (AV 3.45±0.61; AV+S 2.09±0.26; AV+S+HC 2.58±0.37; AV+S+IC 2.30±0.32 mean ratio ipsi-/contralateral ± SE).

The real-time PCR analysis was performed to measure GFAPmRNA in the RNA in all ventral horn after avulsion. Figure 4 demonstrated that the levels of GFAPmRNA were similar in all groups, 1 week (Fig 4 J) after lesion (AV 1.51±0.38; AV+S 1.20±0.24; AV+S+HC 1.65±0.26 mean ratio ipsi-/contralateral ± SE). However, 4 weeks (Fig 4 K) after avulsion, AV+S group presented a decreased number of transcripts to GFAP (AV 2.64±0.45; AV+S 1.35±0.14; AV+S+HC 2.11±0.12; AV+S+IC 2.00±0.12 mean ratio ipsi-/contralateral ± SE).

### 3.4- Microglial reactivity is enhanced by mononuclear cells one week after tranplantation

**Figure 5:**
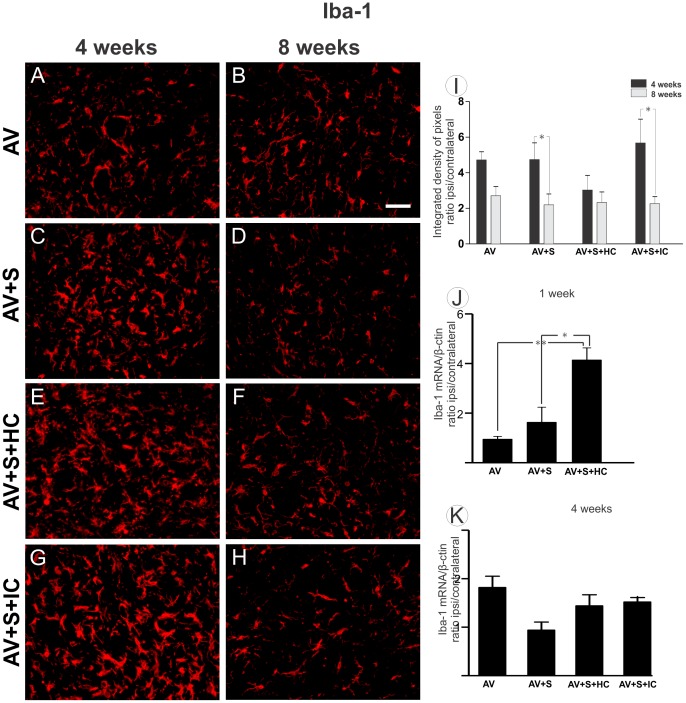
Microglial analysis of the spinal cord ventral horn 4 and 8 weeks after VRA. The quantification analysis on motoneuron cell bodies stained with anti- Iba1 of the ipsilateral side of VRA after 4 weeks (A, C, E and G) and after 8 weeks (B, D, F and H). (A and B) AV, (C and D) AV+S, (E and F) AV+S+HC and (G and H) AV+S+IC. Scale bar  = 50 µm. (I) The mean ratio of the ipsil-/contralateral integrated intensity of pixels of the ipsilateral and contralateral sides in both groups. Note the decrease in microglial reactivity in AV+S and AV+S+IC as compared 4 and 8 weeks after injury. (J) There were significant increase in the synthesis of Iba-1 mRNA in the lumbar spinal cord one week after avulsion (*p<0.05 and **p<0.01, n = 5). (K) The RT-qPCR, performed 4 weeks after avulsion, did not demonstrate significant differences between groups in Iba-1 mRNA.

To detect possible changes in the microglial cells close to large motoneurons cell bodies after avulsion, the immunoreactivity against Iba-1 was evaluated in the ventral horn in the different experimental groups. Figure 5 shows that the microglial reactivity was not significantly further increased after implantation and cell treatment in both experimental times. However, the AV+S and AV+S+IC groups showed decreased microglia reaction in the course of experimental time. Four weeks after avulsion: (AV 4.71±0.41; AV+S 4.73±0.94; AV+S+HC 3.02±0.64; AV+S+IC 5.67±1.34; mean ratio ipsi-/contralateral ±SE with p<0.01) and 8 weeks after avulsion: (AV 2.70±0.51; AV+S 2.18±0.57; AV+S+HC 2.32±0.59; AV+S+IC 2.25±0.35 mean ratio ipsi-/contralateral ± SE, p<0.05).

The real-time PCR analysis was performed to measure Iba-1 mRNA in the in ventral horn RNA after avulsion. Figure 5 demonstrated that the levels of Iba-1 mRNA in AV+S+HC were significantly greater than in the AV and AV+S groups (AV 0.95±0.30; AV+S 1.63±0.60; AV+S+HC 4.15±0.48 mean ratio ipsi-/contralateral ± SE). Nevertheless, 4 weeks (Fig 5 K) after avulsion, Iba-1 mRNA were similar in all groups (AV 2.07±0.26; AV+S 1.07±0.18; AV+S+HC 1.64±0.26; AV+S+IC 1.73±0.03 mean ratio ipsi-/contralateral ± SE).

### 3.5- Mononuclear cells enhance BDNF and GDNF expression one week after avulsion

**Figure 6:**
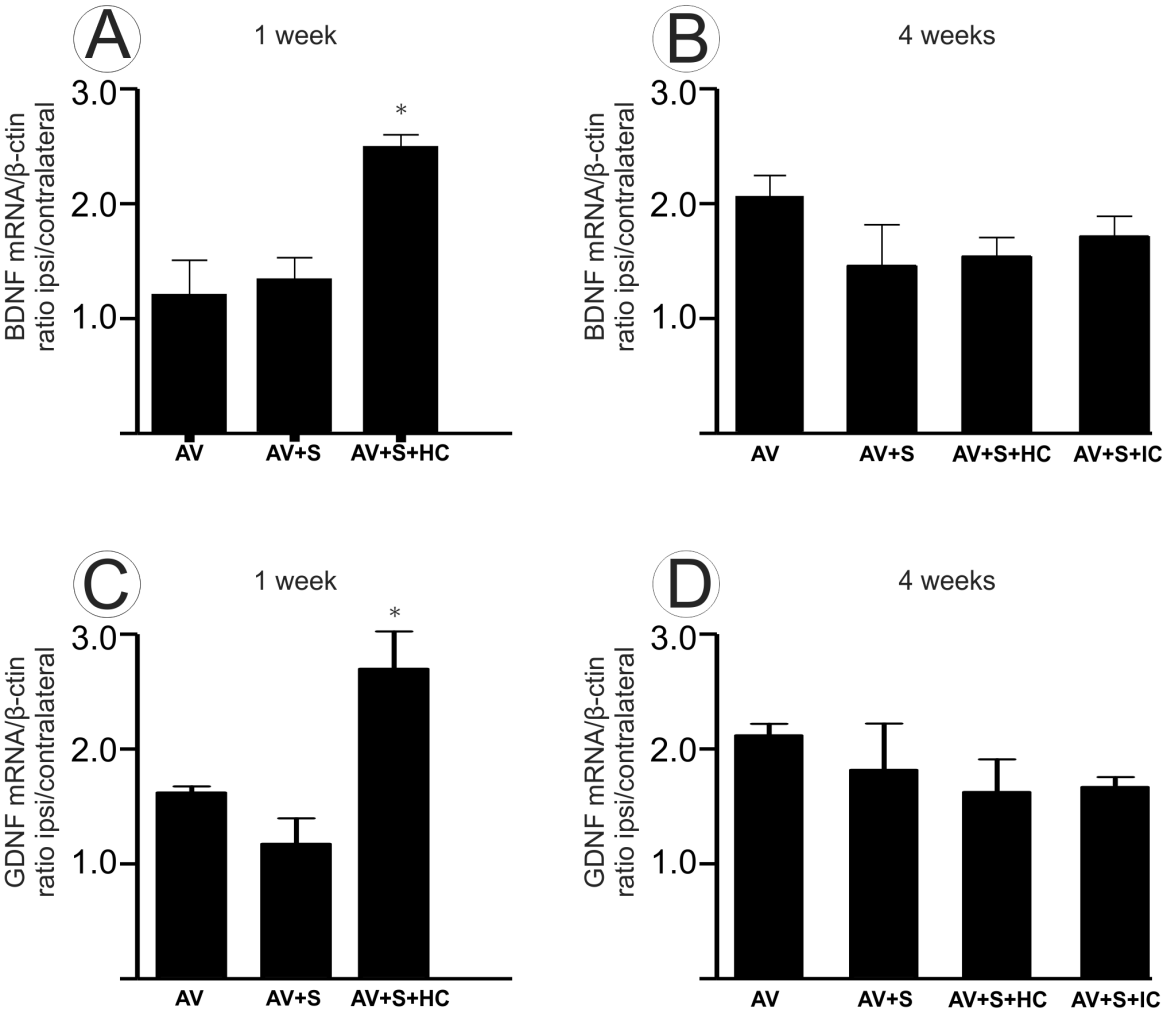
Neurotrophic factor expression (BDNF and GDNF) by RT-qPCR in the ventral horn spinal cord 1 and 4 weeks after avulsion. Expression of mean ratio of the ipsi-/contralateral mRNA for BDNF obtained by RT-qPCR in the lumbar spinal cord one (A) and four (B) weeks after avulsion. Note that one week after avulsion AV+S+HC enhanced the BDNF mRNA production compared to others groups (*p<0.05, n = 5). Expression of mean ratio of the ipsi-/contralateral mRNA for GDNF obtained by RT-qPCR in the lumbar spinal cord one (C) and four (D) weeks after avulsion. Note that, one week after avulsion in the AV+S+HC group, there is enhanced GDNF mRNA production as compared to others groups (*p<0.05, n = 5).

Real time PCR was used to detect BDNF and GDNF mRNAs in the lumbar spinal cord after avulsion (Figure 6). The results demonstrated the levels of both neurotrophic factors in the AV+S+HC group were significantly greater, when compared to the other groups, one week after surgery (A and C) (BDNF - AV 1.20±0.29; AV+S 1.33±0.18; AV+S+HC 2.48±0.10 mean ratio ipsi-/contralateral ± SE *p<0.05) and (GDNF - AV 1.71±0.56; AV+S 1.24±0.23; AV+S+HC 2.86±0.34; mean ratio ipsi-/contralateral ± SE *p<0.05). Four weeks after avulsion, BDNF and GDNF mRNA levels were similar in all groups (B and D) (BDNF - AV 2.04±0.18; AV+S 1.45±0.35; AV+S+HC 1.53±0.16; AV+S+IC 1.70±0.17 mean ratio ipsi-/contralateral ± SE) and (GDNF - AV 2.25±0.10; AV+S 1.92±0.43; AV+S+HC 1.72±0.30; AV+S+IC 1.77±0.01 mean ratio ipsi-/contralateral ± SE).

### 3.6- Functional motor recovery

**Figure 7:**
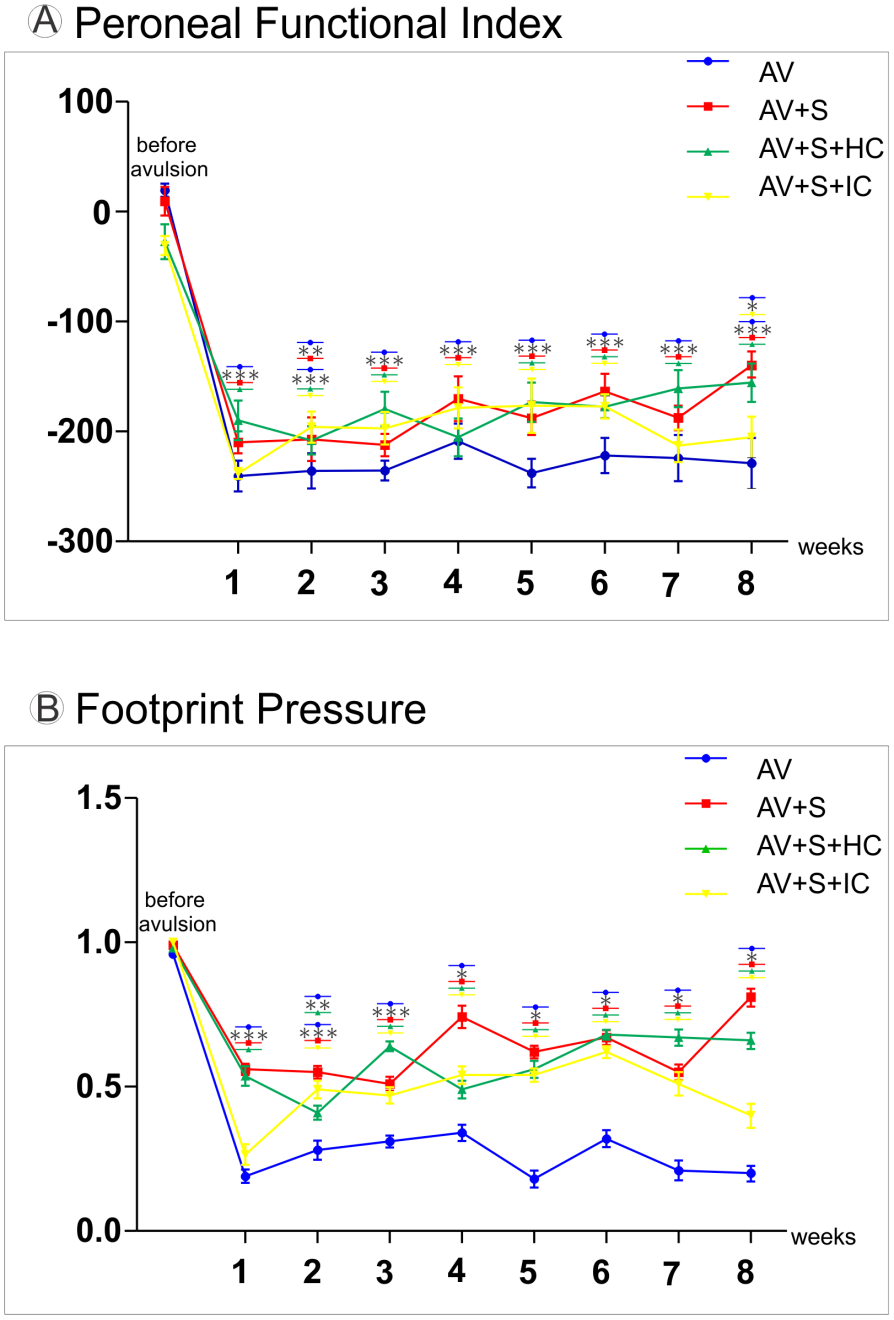
Motor function recovery after ventral root treatment. (A) Graph of the peroneal nerve functional index up to 8 weeks after avulsion. There is a significantly better performance of the three implanted groups compared to AV from the first week post lesion until the eighth week (***p<0.001, **p<0.01 and *p<0.05 n = 10). (B) Restoration of weight-bearing capacity following avulsion. There is also a restoration of weight-bearing capacity following avulsion in implanted groups with or without cells from the first up to the eight week after injury. Values are expressed as the ratio of ipsi-/contralateral pressure exerted by the paw on the catwalk platform (***p<0.001 and *p<0.05, n = 10).

The recovery of motor function was analysed by the CatWalk System. Post-operative assessments of peroneal function were performed for eight consecutive weeks. The preoperative peroneal functional index mean values (Figure 7A) did not significantly differ between groups. The peroneal functional index drastically declined after avulsion. The three implanted groups presented significantly better peroneal index performance as compared to AV group (AV −228.89±23.0; AV+S −139.75±12.5; AV+S+HC −155.50±17.2; AV+S+IC −205.14±18.6, mean ratio ipsi-/contralateral ± SE, *p<0.05, **p<0.01 and ***p<0.001. These results are consistent with the footprint paw pressure data (Figure 7B), indicating that all three implanted groups presented a better performance as compared to avulsion group (AV 0.20±0.07; AV+S 0.81±0.09726454; AV+S+HC 0.66±0.078; AV+S+IC 0.40±0.11 mean ratio ipsi-/contralateral ± SE with *p<0.05 and ***p<0.001).

## Discussion

Brachial plexus lesions are particularly debilitating and usually affect young adults. To date, new technological refinements aiming at improving the repair of such traumatic injury are necessary. Although nerve reimplantation has been proposed [7], [10] the relatively poor clinical outcome requires further attention. Treatment with neurotrophic factors, following root reimplantation, has proven to be efficient [17]. Importantly, it has been previously shown that mesenchymal stem cells naturally produce BDNF and GDNF when grafted to the VRA lesion area [18]. Considering it, the present study main objective was to compare the root reimplantation regenerative outcome by either injecting or engrafting the mononuclear cells at the wounded area, with aid of a fibrin sealant scaffold. However, evaluating the perspective of translational studies, the use of bone marrow MC has been preferred instead of mesenchymal stem cells.

The use of MC resulted in neuroprotection following peripheral nervous system lesion [1], [3] and following spinal cord injury [28]. Furthermore, the mononuclear fraction is easy to obtain, implicating in lower costs and less processing time, as compared with other cell types [1], [29].

An important point to be observed is the best moment for performing the cell therapy. Therefore, in some instances, it has been proposed a late cell transplantation, following the acute phase after injury [30]. However, immediate cell engrafting increases the survival of motoneurons after root avulsion [17].

Based on such observations, the present work was based on cells treatment immediately after avulsion, in order to obtain the best neuroprotective results possible. This is particularly important following CNS/PNS injuries that cause extensive degeneration of adult motoneurons [31]–[33]. Such neuronal loss reaches up to 80% of motoneuron degeneration in the first two weeks post injury [7]. Our results showed that both intraspinal administration and fibrin sealant implantation of MC preserved a significant number of motoneurons when compared to the untreated group (avulsion). The neuroprotective effect observed herein reinforces that the combination of root reimplantatio and cell therapy leads to enhancement of axotomized motoneurons. On the contrary, most of the motoneurons degenerated following AV, which reinforces the need of acute neuroprotective treatments following VRA.

Neuronal rescue was highest following root reimplantation and fibrin sealant cell engrafting, 4 weeks after injury. Such effect may be the result of stem cell neurotrophic factor release in the site of injury [17], [18]. Nevertheless, the production of extracellular matrix molecules has also been suggested as neuroprotective [34]. Neuronal survival decreased at 8 weeks post injury, indicating that the therapeutic window for MC is relatively short and further cell treatments may be necessary to maintain the axotomized neurons.

Cell injection to the spinal cord gave similar results to the reimplantation alone, 4 weeks post injury. However, neuronal degeneration increased up to 8 weeks, reaching the level of avulsion only. We believe that the injection into the CNS/PNS interface result in a late inflammatory response that cause delayed cell death. In this sense, using a similar reimplantation approach, followed by mesenchymal stem cell injection, neuron preservation has been shown without significant functional recovery [35]. Overall, the neuronal survival results indicate that fibrin sealant homogenized MC result in acutely better results as compared to intraspinal engrafting.

According to prior studies, MC express BDNF and GDNF [3] in the PNS following sciatic nerve injury. BDNF is also produced by MCs in the CNS, 3 and 7 days after MC therapy [4]. In line with this, several studies have demonstrated neuroprotective effects of such neurotrophic factors [7], [32], [36], especially after spinal cord injury [37]. Neuroprotection is also achieved in neonatal animals [38], where the effects of axotomy are more drastic than in adults. The results herein are in line with the literature and show BDNF and GDNF production by the MC, 1 week post injury.

Interestingly, BDNF, which is exacerbated in the implant group, promotes axonal sprouting and elongation after peripheral nerve injury and during development [39]. This is in line with the motor recovery observed in all root reimplanted groups, suggesting regeneration of axons after avulsion.

Functional analysis of the peroneal nerve, as well as the pressure exerted by the ipsilateral paw, provides reliable data on functional recovery of animals with motor problems [40], [41]. The motor improvement observed herein is noteworthy because gait restoration is necessarily related to motor units recovery, together with cerebral cortex control [42]. Importantly, the preservation of pre-synaptic inputs to the injured motoneurons after implantation, observed by immunohistochemistry, indicates preservation of supraspinal contacts.

An important occurrence following VRA is the reduction of pre-synaptic terminals to motoneurons [42], [43]. This synaptic decrease become irreversible if the regrowing axons within the root do not reach their target muscles [43]. Importantly, the groups subjected to root reimplantation showed a significant preservation of synapses as compared to the avulsion only group. In this case, cell treatment gave no further benefit, indicating that the restoration of CNS/PNS connection is sufficient to preserve spinal synaptic networks. Coupled with such synaptic changes, glial cells became reactive following VRA. Such reactive gliosis is characterized by hypertrophy of the cell body and processes of astrocytes and microglial hyperplasia [44]–[46].

Although the microglial reaction was morphologically decreased by MC treatment four weeks post lesion, at one week, an enhancement of Iba-1 gene transcripts was obtained. One possibility is that MCs increased or accelerated acute microglial response [47]. Regarding the astrogliosis, no differences were perceived between groups by immunohistochemical analysis. qPCR data, on the other hand, indicated a decreased number of GFAP gene transcripts following root reimplantation alone. This is in line with what has been observed 12 weeks after root reimplantation [10].

## Conclusions

Taken together, the results of the present study indicate that MCs therapy further preserves injured motoneurons up to 4 weeks after implantation, in comparison to root reimplantation alone. Such fact is possibly related to the production of neurotrophic factors, such as BDNF and GDNF. Nonetheless, such difference was not seen at 4 weeks post injury. This may in turn indicate that restoration of the CNS/PNS connection must be carried out in a short period of time after avulsion. Importantly, the present data indicate that cell injection to the spinal cord does not result in long lasting neuroprotection. On the other hand, engrafting of MC at the site of injury with the aid of a fibrin scaffold is effective and may represent a more practical approach, with regard to translational medicine.
